# Biochemical and biological characterization of exosomes containing prominin-1/CD133

**DOI:** 10.1186/1476-4598-12-62

**Published:** 2013-06-14

**Authors:** Germana Rappa, Javier Mercapide, Fabio Anzanello, Robert M Pope, Aurelio Lorico

**Affiliations:** 1 Cancer Research Center, Roseman University of Health Sciences, Las Vegas, NV, 89135, USA; 2 Department of Medical Administration, University of Iowa Carver College of Medicine, Iowa City, Iowa, USA

**Keywords:** Exosomes, Melanoma, Prominin-1, Proteomics, Lipidomics, micro-RNA

## Abstract

Exosomes can be viewed as complex “messages” packaged to survive trips to other cells in the local microenvironment and, through body fluids, to distant sites. A large body of evidence indicates a pro-metastatic role for certain types of cancer exosomes. We previously reported that prominin-1 had a pro-metastatic role in melanoma cells and that microvesicles released from metastatic melanoma cells expressed high levels of prominin-1. With the goal to explore the mechanisms that govern proteo-lipidic-microRNA sorting in cancer exosomes and their potential contribution(s) to the metastatic phenotype, we here employed prominin-1-based immunomagnetic separation in combination with filtration and ultracentrifugation to purify prominin-1-expressing exosomes (prom1-exo) from melanoma and colon carcinoma cells. Prom1-exo contained 154 proteins, including all of the 14 proteins most frequently expressed in exosomes, and multiple pro-metastatic proteins, including CD44, MAPK4K, GTP-binding proteins, ADAM10 and Annexin A2. Their lipid composition resembled that of raft microdomains, with a great enrichment in lyso-phosphatidylcholine, lyso-phosphatidyl-ethanolamine and sphingomyelin. The abundance of tetraspanins and of tetraspanin-associated proteins, together with the high levels of sphingomyelin, suggests that proteolipidic assemblies, probably tetraspanin webs, might be the essential structural determinant in the release process of prominin-1 of stem and cancer stem cells. Micro-RNA profiling revealed 49 species of micro-RNA present at higher concentrations in prom1-exo than in parental cells, including 20 with cancer-related function. Extensive accumulation of prom1-exo was observed 3 h after their addition to cultures of melanoma and bone marrow-derived stromal cells (MSC). Short-term co-culture of melanoma cells and MSC resulted in heterologous prominin-1 transfer. Exposure of MSC to prom1-exo increased their invasiveness. Our study supports the concept that specific populations of cancer exosomes contain multiple determinants of the metastatic potential of the cells from which they are derived.

## Introduction

Development of effective anti-cancer strategies based on prevention and targeting of metastatic disease is of high priority, particularly for melanoma, a disease for which the development of metastasis is by far the major cause of patients’ death [1]. Tumor-derived exosomes, small extracellular vesicles that perform diverse cellular functions including intercellular communication, antigen presentation, and transfer of proteins, RNA and lipids, have been recently implicated in the metastatic process. Exosomes originate by a sequential process of inward budding of late endosomes, producing multivesicular bodies (MVBs), followed by release of internal microvesicles into the microenvironment by fusion of the MVBs with the plasma membrane [2]. Cancer exosomes may have a role in the cross-talk between primary tumors and bone marrow-derived stromal cells (MSC), reprogramming MSC and other non-tumor cells to support local cancer growth as well as to prime pre-metastatic niche(s) [3-7]. However, difficulties in obtaining homogeneous exosomal preparations result in incomplete understanding of exosome formation, composition and functions [8]. We recently reported a novel identification of the extracellular release of prominin-1-containing membrane microvesicles from human FEMX-I metastatic melanoma cells, and suggested that prominin-1 microvesicles influence the metastatic capacity of FEMX-I cells [9]. Our laboratory had previously shown that prominin-1 knock-down resulted in decreased metastatic potential of FEMX-I cells in immune-deficient mice [10]. Prominin-1, a pentaspanning transmembrane protein originally identified as a surface marker of both neural [11] and hematopoietic [12] stem and progenitor cells, is expressed in both established melanoma cell lines and clinical specimens derived from melanoma patients [13-16].

Here, we have employed immune-selection forprominin-1 to isolate and characterize a homogenous preparation of exosomes, presumably engineered from FEMX-I melanoma cells to perform unique and pro-metastatic tasks in the local microenvironment.

### Experimental procedures

#### *Cell Culture*

The human FEMX-I cell line was originally derived from a lymph node metastasis of a patient with malignant melanoma [17]. Cells were routinely cultured in RPMI (Mediatech Inc., Manassas, VA, http://www.cellgro.com) supplemented with 10% fetal bovine serum (FBS) (Atlanta Biologicals, Lawrenceville, GA, http://www.atlantabio.com) at 37°C in a 5% CO_2_ humidified incubator and used between passages 3 and 15. Human MSC were obtained from Dr. Prockop, Texas A & M. They were isolated from 1 to 4-ml bone marrow aspirates taken from the iliac crest of normal adult donors after informed consent and under a protocol approved by the Texas A & M Institutional Review Board, prepared as described by Larson et al. [18], and frozen at passage 1. For expansion, MSC were plated in a 75-cm^2^ culture dish, and incubated for 1 day, to recover viable adherent cells. Cultures contained approximately 50% of rapidly self-renewing cells (RS) and 50% of larger, more slowly dividing and more mature cells (MS). MSC were then replated at 50 cells per cm^2^ and incubated for 10 days before lentiviral transduction. With time in culture, the percentage of RS cells decreased progressively to less of 10% of the total cells. All cell lines were stored in aliquots in liquid nitrogen and kept in culture for less than 3 months. Complete culture medium for MSC consisted of α-minimal essential medium (Gibco, Grand Island, NY), 17% fetal bovine serum (lot selected for rapid growth of MSC) (Atlanta Biologicals), 100 units/ml penicillin, 100 μg/ml streptomycin, and 2 mM L-glutamine. Cells were routinely tested for mycoplasma contamination by the Venor GeM mycoplasma detection kit (Sigma-Aldrich, St. Louis, MO) and by DAPI staining and authenticated by morphology check every two weeks.

#### *Preparation of microvesicles and exosomes*

For preparation of FEMX-I microvesicles (“classical” preparation) and exosomes (prom1-exo), cells were enzymatically detached and cultured for six days as spheroids in serum-free medium, consisting of Dulbecco’s modified Eagle’s medium in the presence of B27 supplement (both from Gibco) in tissue culture plates, as previously described [9]. At time of harvest, the pH of the medium was 6.7. “Classical” microvesicle preparations were performed by differential centrifugation at 4°C at 300 × g for 5 min, then at 500 × g for 5 min., at 1,200 × g for 20 min. and at 10,000 × g for 30 min, followed by centrifugation at 200,000 × g for 60 min at 4°C. Because these preparations are likely to contain a mixture of both exosomes and other microvesicles, we have used the generic term microvesicles in this study to include the exosome pool. Prom1-exo were prepared by differential centrifugation at 4°C at 300 × g for 5 min, then at 500 × g for 5 min., at 1,200 × g for 20 min. and at 10,000 × g for 30 min, followed by filtration with a 0.22 μm low-protein binding Millex-GV filters (Millipore); the 10,000 × g supernatant was then concentrated by Amicon Ultracel-100K (Millipore) tubes according to the manufacturer’s instructions. The concentrate was diluted 1:1 (v/v) with PBS; incubated with anti-IgG microbeads (Miltenyi Biotec, Auburn, CA) for 90 min at 4°C, and passed through LS-columns according to the manufacturer’s instructions. The flow-through was collected, incubated with anti-human-prominin-1 beads (Miltenyi) for 1 h, and passed through LS-columns. After washings, the column was removed from the magnet and prom1-exo were flushed down with 10 ml of cold PBS. Prom1-exo were then centrifuged at 200,000 g for 60 min at 4°C and resuspended in PBS. Each exosomal preparation was checked by nanoparticle tracking analysis for size distribution and microparticle concentration and by Western blotting for expression of prominin-1. Exosomes and microvesicles were stained with PKH67 (Sigma-Aldrich, St. Louis, MO), according to the manufacturer’s protocol.

#### *Nanoparticle tracking analysis (NTA)*

We used the light-scattering characteristics of 488 nm laser light on microvesicle preparations undergoing Brownian motion injected by continuous flow into the sample chamber of an LM10 unit (Nanosight, Amesbury, UK). Three videos of 60–90 seconds were recorded of each sample. Data analysis was performed with NTA 2.3 software (Nanosight). The diffusion coefficient and hydrodynamic radius were determined using the Stokes–Einstein equation, and results were displayed as a particle size distribution. Data are presented as the average and standard deviation of the three video recordings. Since NTA is most accurate between particle concentrations in the range of 2 × 10^8^ to 2 × 10^9^/ml, when samples contained higher numbers of particles, they were diluted before analysis and the relative concentration calculated according to the dilution factor. Control 100 and 200 nm beads were supplied by Nanosight. NTA of a small sample of any given preparation revealed that they were essentially monodisperse, excluding the problem of aggregation, which may significantly impact on a biological system.

#### *Prominin-1-EGFP fusion plasmid and transfection*

We employed the eukaryotic expression plasmid enhanced GFP (pEGFP)–N1-prominin-1, containing the entire coding sequence of human prominin-1 fused in-frame to the N-terminus of GFP [19], to transfect FEMX-I cells, as previously described [9].

#### *Protein processing and LC-MS/MS*

##### *Electrophoresis*

Three independent preparations of prom1-exo were analyzed by LC-MS/MS. Samples, 5 μg each according to results of Bradford assays, were individually mixed with 20 μl LDS buffer, divided into four fractions and loaded on NuPage 4–12% Bis-Tris precast gels (Invitrogen, Carlsbad, CA). Two exterior lanes were loaded with Sharp pre-stained protein ladder standards (Invitrogen) and the gel was electrophoresed according the manufacturer’s recommendations. Two lanes, one containing Sharp prestained standards and one containing one fourth of the total sample, were visualized using a silver nitrate protocol (QuickSilver, Pierce, Madison, WI), then realigned with the unstained gel section to create a template for excision. The three remaining, unstained lanes were segmented into 14 equal sections and subjected to in-gel tryptic digestion following the procedure of Shevchenko *et al*. [20]. Briefly, the protocol calls for reduction with 10 mM DTT and alkylation with 55 mM iodoacetamide (SIGMA-Aldrich, St. Louis, Mo). Each segment was prepared with successive wash and dehydration steps using 50 mM ammonium bicarbonate (AmBic) or 50% acetonitrile containing 50 mM AmBic, respectively. Finally, the shrunken gel segments were rehydrated with ice cold AmBic containing 12 ng/ml sequencing grade trypsin (Promega, Madison, WI), and allowed to swell on ice for three hours. Digestions were then carried out for 16 h at 57°C. The quality of the digested supernatant was determined prior to lyophilization by spotting 1 μl aliquots mixed 1:1 with a saturated solution of alpha-cyano-4-hydroxycinnamic acid (CHCA) acid in 0.1% trifluroacetic acid (Pierce) and 50% acetonitrile onto a stainless steel target plate with subsequent MALDI/TOF analysis on a Autoflex III TOF/TOF (Bruker, Billerica, MA). The remainder of the gel extract was diluted prior to loading on home-brew StageTips desalting microtip using as previously described[21]. Material eluted below 50% acetonitrile was lyophilized and the concentrated peptides were rehydrated in 15 μL of 0.1% formic acid with 5% LC/MS-grade acetonitrile and 4uL was used for each LC injection.

##### *LC-MS/MS analysis*

Using a Dionex 3000 nanoRSLC series HPLC system (Thermo-Electron, Waltham, MA) recovered peptides were loaded at 2 μl/min onto a 200 μm id by 2.5 cm precolumn (New Objective, Woburn, MA) packed with 5 μm YMC ODS-C18 beads (Waters, Milford, MA). Following an on-line desalting step, trap flow was rerouted through a self-packed 75 um id × 9 cm analytical column containing 3 μm Halo solid-core C-18 particles with 300 Angstrom pore size. A distal spray opening 8 to 10 microns in diameter restricted the hand-packed column. A linear gradient from 95% buffer A [0.1% formic acid, 5% acetonitrile and 94.9% LCMS grade water] to 55% buffer B [90% ACN, 9.9% water and 0.1% FA] was delivered at 200 μl/min over 70 min using a second nano-capacity pump. Following this, the composition of buffer B was ramped to 80% over 5 min, maintained for 5 min and finally decreased to 5% over the final 10 min.

LC effluent was directed to the electrospray source of a linear ion-trap mass spectrometer (LTQ/XL, Thermo-Electron, USA). MS/MS spectra were acquired in a data-dependent acquisition mode that automatically selected and fragmented the five most abundant peaks from each MS spectrum. MS.MS scans were recorded in centroid mode targeting 8000 counts. The trap was filled for a maximum of 10 ms prior to isolation of the target peptide at an average value 1E04.

##### *Database searching*

Tandem mass spectra were processed and charge states ascertained without deisotoping by Mascot Distiller version 2.4. All MS/MS samples were analyzed using batch processing with the Mascot Daemon interface (version 2.4, Matrix Science) and MASCOT search engine (version 2.4 Matrix Science) [21]. All spectral files were also searched using Spectrum Mill Proteomics Workbench (Rev.Rev A.03.02.060, Agilent Technologies, Santa Clara, CA) and X! Tandem (The GPM, thegpm.org; version CYCLONE (2010.12.01.1)). All three engines were set up to search SwissProt_2012_09.fasta (selected for Homo sapiens, Nov. 24 2012, 20,235 entries) assuming the digestion enzyme trypsin and considering up to two missed cleavages. X! Tandem searches were restricted to the subset of proteins assigned with either Mascot or Spectrum Mill.

Mascot, Spectrum Mill and X! Tandem were searched with a fragment ion mass tolerance of 0.40 Da and a parent ion tolerance of 1.8 Da. Mascot’s Carbamidomethylation of cysteine was specified in Mascot and X! Tandem as a fixed modification. Oxidation of methionine, carbamidomethylation of lysine were specified in X!Tandem and Mascot as variable modifications. Oxidation of methionine was the only variable modification specified in SpectrumMill.

##### *Criteria for protein identification*

Scaffold (version Scaffold_4.0.0, Proteome Software Inc., Portland, OR) was used to validate MS/MS based peptide and protein identifications. Peptide identifications were accepted if they could be established at greater than 90.0% probability by the Peptide Prophet algorithm [22]. Protein identifications were accepted if they could be established at greater than 99.0% probability and contained at least 4 identified peptides. The Protein Prophet algorithm as implemented in Scaffold_4.0.0, assigned protein probabilities [23]. Proteins that contained similar peptides and could not be differentiated based on MS/MS analysis alone were grouped to satisfy the principles of parsimony. Specifically the fragmentation patterns of distinct peptides from families of homologous proteins were inspected manually using the protocol described by Tabb et al. [24]. Hence, validating at least four unique peptides for each protein listed individually minimized protein ambiguity. Peptide False Discovery Rates (FDR) were also estimated using Target:Decoy search as described by Elias and Gygi [25,26], with FDR = 2 × (no. of PSM in the decoy)/(No. of all PSM), where PSM are the peptide spectral matches with better than 90% probability as described above. The FDR calculated by this approach, 0.1%, likely benefits from probabilistically merging multiple search algorithms [23].

### Immunoblotting

For immunoblotting, microvesicles and prom1-exo resuspended in PBS were checked for consistency by NTA. Aliquots of microvesicles, exosomes and FEMX-I total cell lysates containing 1–10 μg of protein were mixed 1:1 with SDS sample buffer (NuSep, Bogart, GA) containing 2% 2-mercaptoethanol, boiled for 5 min, and loaded onto a 8% Tris/Glycine/SDS gel. Electrophoretic separation of proteins was performed at a constant voltage of 120 V for 2 h, and electrophoretic transfer of the proteins into Hybond ECL membrane (GE Healthcare, Waukesha, WI) was carried out at constant amperage (30 mA) for 15 h. The blots were blocked with 5% dry milk in Tris-buffered saline containing 0.05% Tween 20 (pH 7.5), antibody, and incubated with W6B3C1 anti-prominin-1 (Miltenyi Biotec, Auburn, CA), or alix 3A9 clone (Cell Signaling Technology, Danvers, MA) at 1:1000 dilution in TBS-T for 5 h at room temperature. After washing with TBS-T, blots were incubated with IRDye 800CW secondary antibody (Li-Cor Biosciences, Lincoln, NE) in TBS-T (1:20,000) for 45 min at room temperature. Finally, blots were washed with TBS-T, scanned by Odyssey infrared imaging system and analyzed by Odyssey 2.1 application software (Li-Cor Biosciences). Gel band densitometric quantification was performed employing the ImageJ64 software (http://rsbweb.nih.gov/ij).

### ESI-MS/MS lipid profiling

An automated electrospray ionization (ESI)-tandem mass spectrometry approach was used, and data acquisition and analysis were carried out as described previously [27,28] with modifications. The lipid extracts from the FEMX-I cell pellets were dissolved in 1 ml chloroform. An aliquot of 50 μl of each extract in chloroform was used for analysis. Precise amounts of internal standards, obtained and quantified as previously described [29], were added in the following quantities (with some small variation in amounts in different batches of internal standards): 0.6 nmol di12:0-PC, 0.6 nmol di24:1-PC, 0.6 nmol 13:0-lysoPC, 0.6 nmol 19:0-lysoPC, 0.3 nmol di12:0-PE, 0.3 nmol di23:0-PE, 0.3 nmol 14:0-lysoPE, 0.3 nmol 18:0-lysoPE, 0.3 nmol di14:0-PG, 0.3 nmol di20:0(phytanoyl)-PG, 0.3 nmol di14:0-PA, 0.3 nmol di20:0(phytanoyl)-PA, 0.2 nmol di14:0-PS, 0.2 nmol di20:0(phytanoyl)-PS, and 0.23 nmol 16:0–18:0-PI. The sample and internal standard mixture was combined with solvents, such that the ratio of chloroform/methanol/300 mM ammonium acetate in water was 300/665/35, and the final volume was 1.4 ml. The microvesicle samples were prepared similarly, except that the entire sample was analyzed, 1/3 of the above standard amounts were added, and the final volume was 0.75 ml. The unfractionated lipid samples with internal standards were introduced by continuous infusion into the ESI source on a triple quadrupole MS/MS (API 4000, Applied Biosystems, Foster City, CA). Samples were introduced using an autosampler (LC Mini PAL, CTC Analytics AG, Zwingen, Switzerland) fitted with the required injection loop for the acquisition time and presented to the ESI needle at 30 μl/min. Sequential precursor and neutral loss scans of the extracts produce a series of spectra with each spectrum revealing a set of lipid species containing a common head group fragment. Lipid species were detected with the following scans: PC and lysoPC, [M + H]^+^ ions in positive ion mode with Precursor of 184.1 (Pre 184.1); PE and lysoPE, [M + H]^+^ ions in positive ion mode with Neutral Loss of 141.0 (NL 141.0); PG, [M + NH_4_]^+^ in positive ion mode with NL 189.0 for PG; PI, [M + NH_4_]^+^ in positive ion mode with NL 277.0; PS, [M + H]^+^ in positive ion mode with NL 185.0; PA, [M + NH_4_]^+^ in positive ion mode with NL 115.0. SM was determined from the same mass spectrum as PC (precursors of m/z 184 in positive mode) [27,30] and by comparison with PC internal standards using a molar response factor for SM (in comparison with PC) determined experimentally to be 0.39. The collision gas pressure was set at 2 (arbitrary units). The collision energies, with nitrogen in the collision cell, were +28 V for PE, +40 V for PC (and SM), +25 V for PA, PI and PS, and +20 V for PG. Declustering potentials were +100 V for all lipids. Entrance potentials were +15 V for PE and +14 V for PC (and SM), PA, PG, PI, and PS. Exit potentials were +11 V for PE and +14 V for PC (and SM), PA, PG, PI, PS. The scan speed was 50 or 100 u per sec. The mass analyzers were adjusted to a resolution of 0.7 u full width at half height. For each spectrum, 9 to 150 continuum scans were averaged in multiple channel analyzer (MCA) mode. The source temperature (heated nebulizer) was 100°C, the interface heater was on, +5.5 kV or -4.5 kV were applied to the electrospray capillary, the curtain gas was set at 20 (arbitrary units), and the two ion source gases were set at 45 (arbitrary units). The background of each spectrum was subtracted, the data were smoothed, and peak areas integrated using a custom script and Applied Biosystems Analyst software, and the data were isotopically deconvoluted. The first and typically every 11^th^ set of mass spectra were acquired on the internal standard mixture only. Peaks corresponding to the target lipids in these spectra were identified and molar amounts calculated in comparison to the two internal standards on the same lipid class, except for PI, which was quantified in relation to a single internal standard. Ether-linked (alk(en)yl,acyl) lipids were quantified in comparison to the diacyl compounds with the same head groups without correction for response factors for these compounds as compared to their diacyl analogs. To correct for chemical or instrumental noise in the samples, the molar amount of each lipid metabolite detected in the “internal standards only” spectra was subtracted from the molar amount of each metabolite calculated in each set of sample spectra. The data from each “internal standards only” set of spectra was used to correct the data from the following 10 samples. Finally, the data were corrected for the fraction of the sample analyzed and normalized to the mg protein to produce data in the units nmol/mg.

### miRNA profiling

The miRNA profiling array was carried out using Applied Biological Materials miRNA profiling service (ABM C201). Total RNA from FEMX-I cells and exosomes was prepared byQiazol extraction followed by poly-A tailing reactions and miRNA cDNA synthesis (ABM C204). 250 ng of cell’s total RNA and exosomes’ RNA were used in cDNA synthesis. Both cells’ and exosomes’ cDNA synthesis were carried out simultaneously and equal volume of cDNA synthesis reaction product was used in the subsequent profiling. The Ct values for each miRNA-specific cDNA were compared between FEMX-I cells and exosomes. Real-time qPCR reactions and instrumental analysis was performed using Roche LightCycler480. Lists of miRNAs were generated by pair-wise comparison of our expression data sets (cells vs exosomes). Differentially expressed miRNAs were analyzed by the Ingenuity Pathway Analysis software (Ingenuity Systems, Redwood City, CA) to identify the biological functions that were most significant to the data sets.

### Immunofluorescence

Cells were seeded on poly-L-lysine coated chamber slides and grown overnight. Following aspiration of media, cells were fixed in 4% paraformaldehyde (PFA), washed with PBS, permeabilized in 0.5% Tween 20 and blocked with goat serum. After washing with PBS, cells were incubated overnight at 4°C with primary antibodies in 1% BSA-PBS, followed by washes and a 45-minutes incubation at room temperature with fluorochrome-labeled secondary antibody in 1% BSA in PBS. Fluorescent cells were analyzed by a CKX41 fluorescence inverted microscope (Olympus, Center Valley, PA).

### Invasion assay

*In vitro* invasion assays were performed in BioCoat invasion chambers holding matrigel-coated-8 μm-pore PET membrane cell culture inserts, using non-coated inserts as control (both from BD Biosciences, San Jose, CA), according to the manufacturer's directions. The matrigel layers of the invasion chambers were rehydrated with serum-free medium. The lower chambers were filled with medium containing 2% FBS, and equal aliquots of cells, pre-incubated for 3 h with or without prominin-1-purified exosomes, were added in serum-free medium to the inserts. Following 24 h incubation at 37°C, the cells on the upper side of the membrane were gently removed with wet sterilized cotton swabs. The cells on the lower surface of the membranes were fixed with 4% para-formaldehyde for 10 min, and then stained with DAPI. The number of cells was counted in 8–12 randomly selected 10X-microscopic fields per insert using an Olympus CKX41 fluorescence microscope (Olympus America Corp., Center Valley, PA), and matrigel invasiveness expressed as the percentage of the number of matrigel-invading cells with respect to the control of chemotactic migration.

## Results

### Exosomal preparation

**Figure 1:**
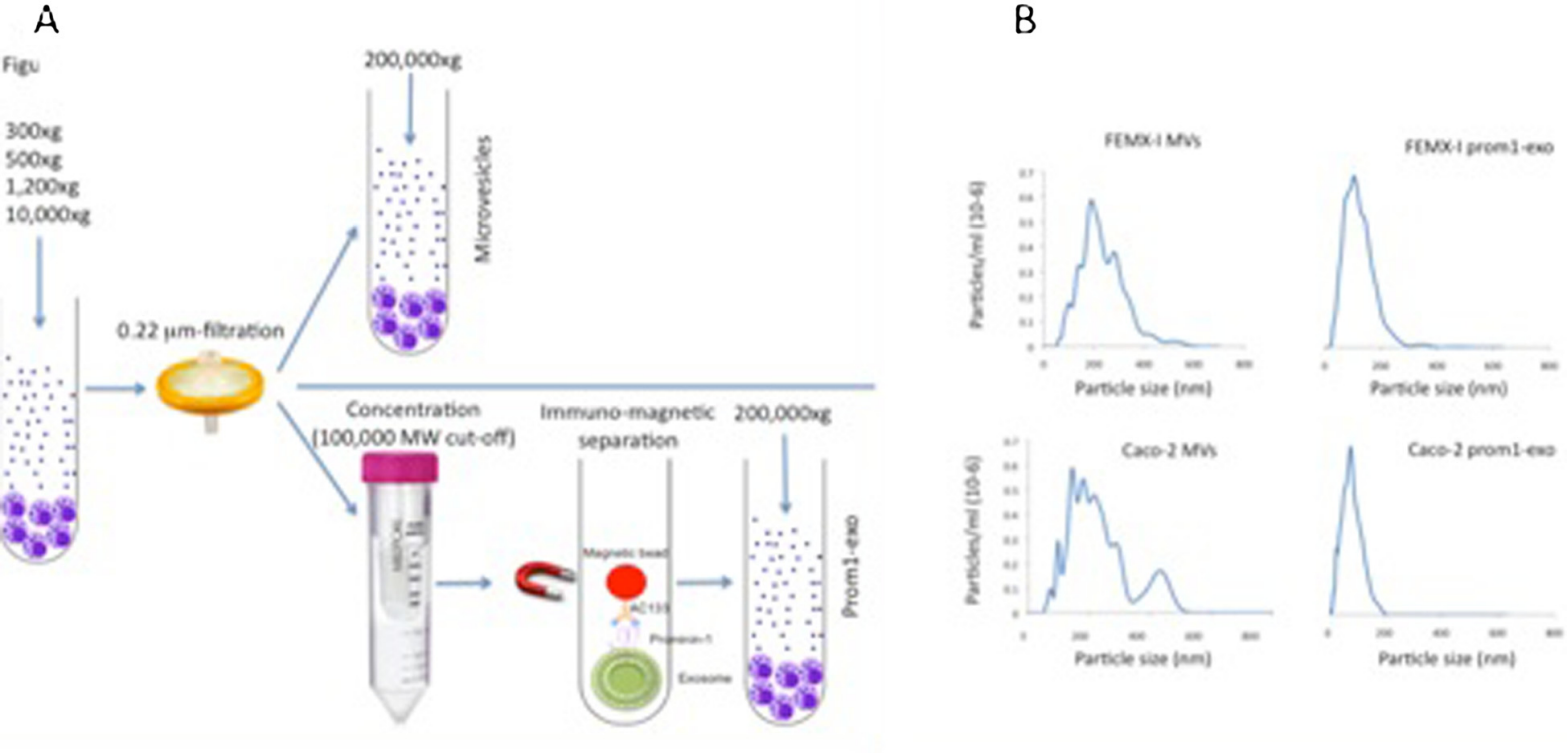
**Isolation and characterization of prom1-exo from FEMX-I cells. A**. Scheme of isolation of “classical” microvesicles by differential centrifugation and of prom1-exo by a combination of differential centrifugation, filtration and immuno-magnetic separation. **B**. Microvesicle tracking analysis shows size distribution of a “classical” ultracentrifugation-based preparation of microvesicles and a prominin-1-based immunomagnetic preparation (prom1-exo), both from serum-free culture medium of the human FEMX-I metastatic melanoma cell line. Both preparations were stained with the membrane dye PKH67 and fluorescence analyzed by a 488 nm laser. Nanotracking analysis gives mean peak intensities of 80 and 120 nm for microvesicles and 90 nm for exosomes, respectively. The persistent binding of magnetic beads (50 nm) to the prominin-1 microvesicles resulted in an over-estimation of their size distribution.

We previously reported that human FEMX-I metastatic melanoma cells released into the extracellular medium prominin-1-expressing microvesicles [9]. To investigate their nature, we cultured FEMX-I cells as spheroids under serum-free conditions for six days and compared a “classical” microvesicle preparation, based on differential centrifugation [9], with a prominin-1^+^ preparation, illustrated in Figure 1A, based on the combination of differential centrifugation, filtration and prominin-1-based immuno-magnetic selection (prom1-exo). The final pH at time of harvest was 6.7, which resembled *in vivo* tumor growth conditions, where low pH condition is a hallmark of tumor malignancy, particularly for malignant melanoma cells, which, differently from normal cells, can survive in an acidic microenvironment [31]. Low pH conditions reportedly increase exosome release and uptake by cancer cells [32]. We used serum-free medium in the present study because serum supplements (such as fetal calf serum) often contain vesicles as well as aggregates of serum proteins, which may interfere with the isolation and characterization of FEMX-I exosomes. By NTA, we determined both size distribution and relative concentration of microvesicles and prom1-exo in the supernatants of FEMX-I cells. As shown by NTA of PKH67-stained microvesicles, the “classical” microvesicle preparation showed several peaks, ranging from 70 to 550 nm, while prom1-exo yielded a single peak of about 100 nm (Figure 1B); persistent binding of anti-prominin-1 50 nm-immunomagnetic beads resulted in an apparent over-estimation of the exosomal size and broadening of the size distribution peak. The concentration of microvesicles and prom1-exo in FEMX-I supernatant were 3 ± 0.4 ×10^9^/ml and 0.35 ± 0.2 × 10^9^/ml, respectively. We then employed the same methodology (Figure 1A) to investigate whether it was possible to isolate prom1-exo from different prominin-1-expressing cancer cell lines. We found that prominin-1-immunomagnetic selection resulted in isolation of cancer exosomes also from human prominin-1-expressing Caco-2 colon carcinoma cells (Figure 1B). An approximately 10-fold difference in concentration of microvesicles and prom1-exo was found also in the cell supernatants of Caco-2 cells (1.5 ± 0.35 × 10^9^/ml and 0.18 ± 0.05 × 10^9^/ml, respectively). Similarly to what we observed in FEMX-I cells, Caco-2 cells microvesicles had a broad size range, while prom1-exo had a single 100 nm-peak.

### Proteome of prominin-1^+^ microvesicles

**Figure 2:**
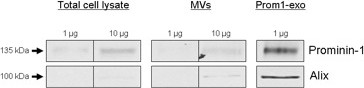
**Enrichment of prominin-1 and alix in prom1-exo.** Immunoblotting analysis of total cell lysates, microvesicles (MVs), and prom1-exo from FEMX-I cells. 1 and 10 μg of total proteins were loaded per lane for total cell lysates and MVs and 1 μg for prom1-exo, and analyzed as described under *Experimental Procedures*.

Comparison of total cell lysates, microvesicles and prom1-exo from FEMX-I cells by Western blotting (Figure 2) revealed a great enrichment in prominin-1 and in the exosomal protein alix in prom1-exo vs. the FEMX-I cells themselves (53- and 184-fold for prominin-1 and alix, respectively) and vs. microvesicles (78- and 168-fold for prominin-1 and alix, respectively). To investigate whether they had the biochemical characteristics of *bona fide* exosomes, we analyzed the proteolipidic composition of prom1-exo from FEMX-I cells. Three independent preparations were used to determine their protein composition via MS/MS mass spectrometry (see search methods in Additional file 1: Table S1). A total of 282 proteins were confidently assigned across all three samples (Additional file 2: Table S2 and Additional file 3: Table S3). We further highlighted an ensemble of proteins among which could be verified with two or more stringent peptide spectral matches (PSM) in all three replicates or those observed with three or more stringent PSM in any two replicates (Additional file 4: Table S4). This subset of 154 proteins is highly enriched for physiological processes (Additional file 5: Figure S1), involving membrane bound vesicles [count 40, p-value 6.4E-21] and endocytosis [count 20, p-value 7.3E-11] complexes, and including all of the 14 proteins most expressed in exosomes according to the compilation of peer-reviewed data hosted on the Exocarta site [33] (Table 1). Since the biogenesis of exosomes takes place in late endosomes to end up in multivesicular bodies (MVB), we first checked our list of proteins for those known to be involved with that particular compartment (Table 2). Reassuringly, we identified the bro1 domain-containing proteins alix and brox, known to function in association with the ESCRT (Endosomal Sorting Complex Required for Transport) pathway to help mediate intraluminal vesicle formation at multivesicular bodies and the abscission stage of cytokinesis. Various ESCRT components, central to MVB biogenesis, were identified in prom1-exo, including five ESCRT-I proteins [34], three ESCRT-III proteins and many other ESCRT-associated proteins (Table 2). Other proteins, related to their endosomal origin, were identified, including membrane transport and fusion proteins (GTPases, Annexin A2, A4, A5, A6 and A11); eight tetraspanins (TSPAN 4,6,9,14; CD63; CD81; CD82; CD9), and five Rab proteins (Additional file 4: Table S4). Interestingly, the immunosuppressive Immunoglobulin superfamily member 8 (IgSF8), also named CD81 partner 3, known to interact with CD81, CD9 and CD82 as well as with integrin alpha-3/beta-1 and integrin alpha-4/beta-1, was highly expressed. The absence of endoplasmic reticulum proteins, such as calnexin and Grp78, and of Golgi proteins, such as GM130, indicated no contamination of vesicles of other compartments in prom1-exo preparations.

**Table 1:** **Prom1**-**exo composition includes all the 14 most**-**expressed exosomal proteins** (**Exocarta**)

**Protein name**	**Gene symbol**	**Accession number**	**Max N**. **of unique peptides**	**Max % coverage**
Heat shock cognate 71 kDa protein	HSPA8	P11142	46	75
CD9 Antigen	CD9	P21926	8	29
Glyceraldehyde-3-phosphate dehydrogenase	GAPDH	P04406	19	66
Actin, cytoplasmic 1	ACTB	P60709	22	74
CD63 Antigen	CD63	P08962	5	22
CD81 Antigen	CD81	P60033	7	32
Annexin A2	ANXA2	P07355	17	52
Alpha-enolase	ENO1	P06733	20	62
Heat shock protein HSP 90-alpha	HSP90AA1	P07900	13	6
Elongation factor 1-alpha 1	EEF1A1	P68104	8	29
Pyruvate kinase isozymes M1/M2	PKM	P14618	21	59
14-3-3 protein epsilon	YWHAE	P62258	6	36
Syntenin-1	SDCBP	O00560	20	86
Programmed cell death 6-interacting protein	PDCD6IP	Q8WUM4	63	75

**Table 2:** **Prom1**-**exo composition includes many ESCRT and ESCRT**-**associated proteins**

**Protein category**	**Gene name**	**Accession number**	**Max N**. **of unique peptides**	**Max % coverage**
ESCRT-I	VPS-28	Q9UK41	9	57
	VPS-37B	Q9H9H4	9	55
	FAM125A	Q96EYS	6	37
	FAM125B	Q9H7P6	5	38
	TSG101	Q99816	13	37
ESCRT-III	CHMP2A	O43633	3	16
	CHMP4B	Q9H444	6	35
	CHMP5	Q9NZZ3	4	31
ESCRT-associated proteins	Brox	Q5VW32	10	40
	PDCD6IP	Q8WUM4	63	75
	VPS-4A	Q9UN37	9	22
	MITD1	Q8VW92	6	36
	IST1	P53990	11	33
	HSPA1A	P08107	17	46
	HSPA8	P11142	46	75

**Figure 3:**
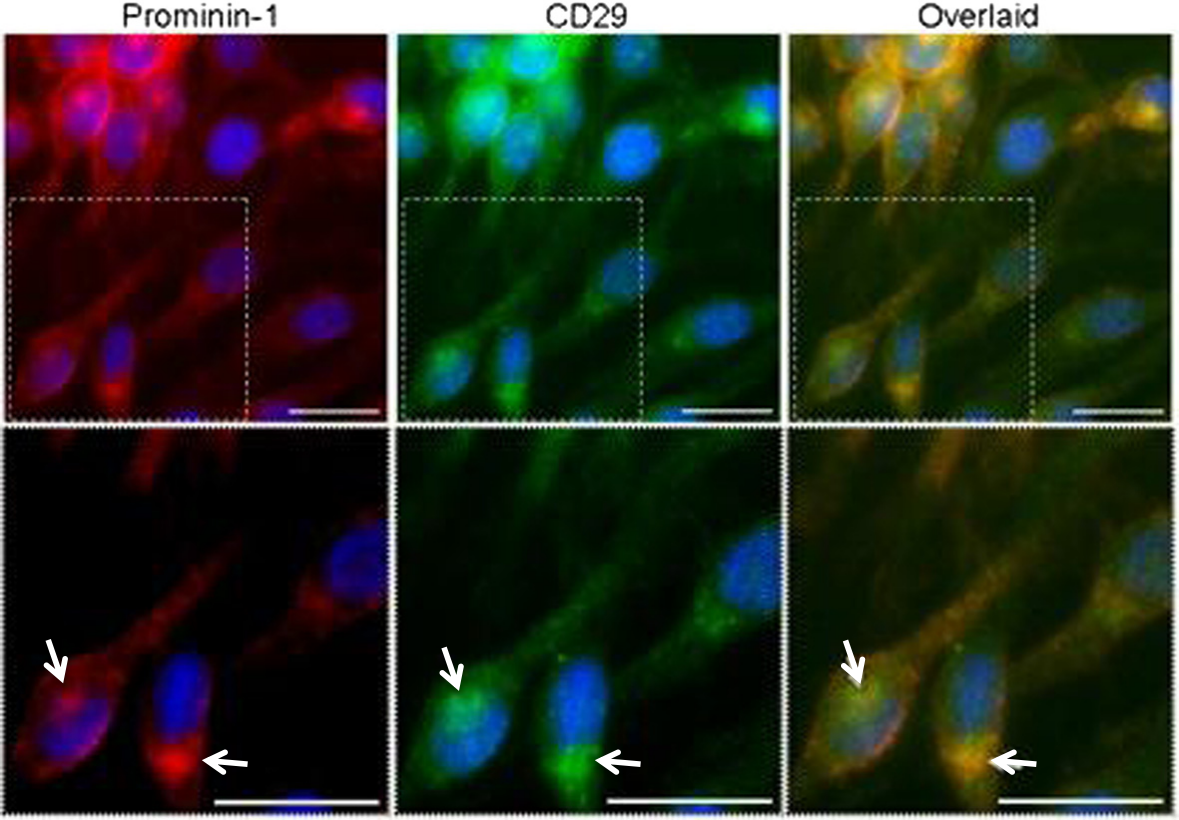
**Co-localization of prominin-1 with CD29 in FEMX-I cells.** Insets in the upper panels were enlarged in the lower panels. Arrows represent areas of peri-nuclear co-localization of prominin-1 and CD29. Prominin-1, red. CD29, green; DAPI, blue. Bars, 25 μm.

Other cancer-related proteins and/or proteins implicated in cancer progression were identified, including CD44 [35], Hsp70 [36], annexin A2 [37-40], as well as components involved in Wnt (SFRP1 = secreted frizzled-related protein 1) and Ras signaling, including the GTP-binding proteins Rap1b and Rap2b, reportedly involved in the activation of ERKs [41], the 14-3-3 protein, a family of exosomal proteins that have a matrix metalloproteinase-1 stimulating effect for dermal fibroblasts [42], and disintegrin and metalloproteinase domain-containing protein 10 (ADAM 10) (Additional file 4: Table S4). A perinuclear pool of prominin-1, associated with integrin-beta 1 (CD29), expressed in FEMX-I exosomes, was detected by fluorescence microscopy (Figure 3). Interestingly, a striking correspondence between prominin-1 and CD29 in FEMX-I cells was observed, suggesting their co-localization in endosomal compartments.

### Prom1-exo have a typical lipid raft composition

**Figure 4:**
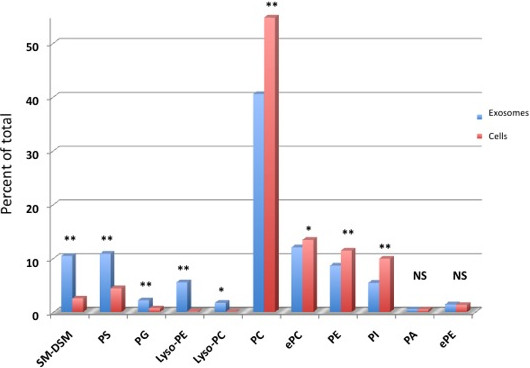
**Different membrane lipid distribution between parental FEMX-I cells and prom1-exo.** An automated ESI-tandem mass spectrometry approach was used. The lipid extracts from cells and microvesicles were dissolved in 1 ml chloroform. An aliquot of 50 μl of each extract in chloroform was used for each analysis. To correct for chemical or instrumental noise in the samples, the molar amount of each lipid metabolite detected in the “internal standards only” spectra was subtracted from the molar amount of each metabolite calculated in each set of sample spectra. The data from each “internal standards only” set of spectra was used to correct the data from the following 10 samples. Finally, the data were corrected for the fraction of the sample analyzed and normalized to the mg protein to produce data in the units nmol/mg. Data are presented as percent of total lipids analyzed. *, p < 0.05; **, p < 0.01 (unpaired Student’s t test). SM-DSM, sphingomyelin-dihydro sphingomyelin; PS, phosphatidylserine; PG, phosphatidylglycerol; e-PE, ether-linked phosphatidylethanolamine; e-PC, ether-linked phosphatidylcholine; PI, phosphatidylinositol; PA, phosphatidic acid.

**Figure 5:**
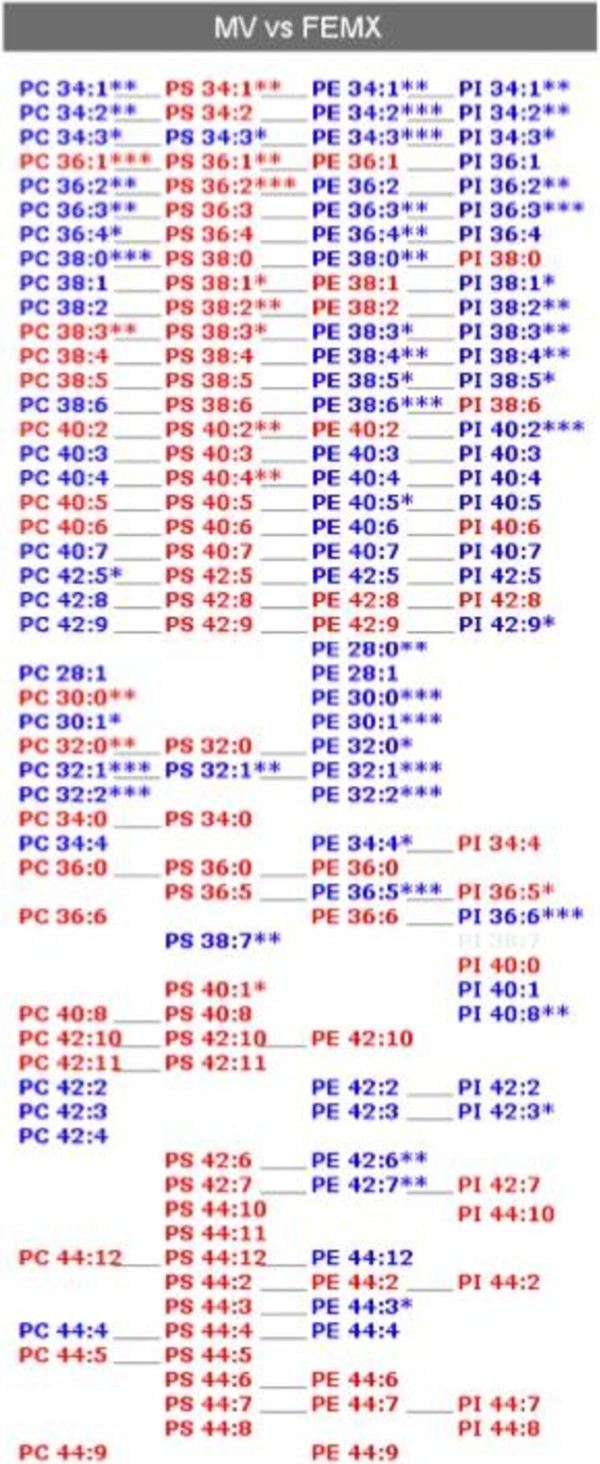
**Differences in lipid profiling between prom1-exo (MV) and parental FEMX-I cells (FEMX).** An automated ESI-tandem mass spectrometry approach was used for lipid profiling. Averages of three to five determinations for each sample group were calculated. Red, lipid species over-expressed in prom1-exo; blue, lipid species over-expressed in parental FEMX-I cells. *, p < 0.05; **, p < 0.01; ***, p < 0.001 (unpaired Student’s t test); head groups: PS, phosphatidylserine; PE, phosphatidylethanolamine; PC, phosphatidylcholine; PI, phosphatidylinositol; the first number indicates the length of the hydrocarbon chain and the second number indicates the number of double bonds.

A lipid composition analysis of prom1-exo and parental FEMX-I cells was performed through ESI MS/MS (Figures 4 and 5). A typical lipid raft composition of prom1-exo was observed, with 400% increase in sphingomyelin, 240% increase in phosphatidylserine, 290% in phosphatidylglycerol, 2150% in lyso-phosphatidylethanolamine) and 1190% in lyso-phosphatidylchoxline. A great number of membrane lipids were significantly different between prom1-exo and the membrane compartment of parental FEMX-I cells (Table 3). To offset the elevated sphingolipid levels, phosphatidylcholine levels were decreased by 26%, resulting in similar choline-containing lipid levels between prom1-exo and the FEMX-I plasma membrane. A 45% decrease in phosphatidylinositol content of prom1-exo also partially accounted for the observed increase in raft-associated lipid species of the exosomes.

**Table 3:** **List of miRNAs over**-**expressed in prom1**-**exo compared with parental FEMX**-**I cells**

**miRNA**	**Ct FEMX-Cells**	**Ct FEMX-Exosomes**	**DCt**	**Fold**-**charge**
hsa-miR-216b	>40	15.81	24.2	19138839.3
hsa-miR-889	37.33	15.86	21.47	2910427.1
hsa-miR-4307	>40	22.51	17.5	184083.4
hsa-miR-4272	>40	22.99	17	131983.7
hsa-miR-203	>40cyclcs	23.82	16.2	74244.7
hsa-miR-4289	23.34	8.67	14.66	25944.3
hsa-miR-3149	22,7	8.77	13.94	15746.0
hsa-miR-203	26.41	13.69	12.72	6769.4
hsa-miR-3145	21.19	10.53	10.66	1622.1
hsa-miR-1911	>40	29.72	10.3	1243.3
hsa-miR-513a-3p	>40	29.84	10.2	1144.1
hsa-miR-3916	>40	30.52	9.48	714.1
hsa-miR-886-3p	>40	32.31	7.69	206.5
hsa-miR-1182	22.77	15.91	6.86	115.1
hsa-miR-3613-5p	>40	33.69	6.31	79.3
hsa-let-7i	22.95	17.21	5.73	53.2
hsa-miR-3132	16.50	11.49	5.01	32.2
hsa-miR-3914	24.75	20.39	4.36	20.5
hsa-miR-3618	28.56	24.35	4.21	18.5
hsa-miR-1307	21.87	17.96	3.91	15.0
hsa-miR-3614-3p	21.90	19.15	2.75	6.7
hsa-miR-519c-3p	22.59	20.22	2.3k	5.2
hsa-miR-3160	17,61	15.28	2.33	5.0
hsa-miR-3153	11.48	9.53	1.96	3.9
hsa-miR-4278	18.94	16.99	1.95	3.9
hsa-miR-3646	I.58	15.80	1.79	3.5
hsa-miR-3926	17.47	15.72	1.75	3.4
hsa-miR-515-5p	28.37	26.69	1.68	3.2
hsa-miR-3169	14.33	12.67	1.66	1.2
hsa-miR-10a	31.87	30.21	1.66	3.2
hsa-miR-140-5p	26.92	25.37	1.55	2.9
hsa-miR-3148	18.74	17.56	1.18	2.3
hsa-miR-4271	17.56	16.48	1.08	2.1
hsa-miR-627	23.07	22.00	1.07	2.1
hsa-miR-548d-3p	29.69	28.66	1.03	2.0
hsa-miR-3613-3p	22.09	21.19	0.90	1.9
hsa-miR-481	26.49	25.64	0.85	1.8
hsa-miR-571	20.81	19.97	0.84	1.8
hsa-miR-4274	19.93	19.15	0.79	1.7
hsa-miR-4277	21.41	20.79	0.62	1.5
hsa-miR-3686	15.41	14.81	0.61	1.5
hsa-miR-3074	21.65	21.10	0.54	1.5
hsa-miR-95	24.90	24.45	0.46	1.4
hsa-miR-590-3p	26.81	26.49	0.32	1.2
hsa-miR-525-5p	23.20	22.90	0.30	1.2
hsa-miR-548g	26.97	26.69	0.28	1.2
hsa-miR-365	25.46	25.18	0.28	1.2
hsa-miR-525-3p	23.23	22.94	0.28	1.2
hsa-miR-320d	21.97	21.93	0.04	1.0

Total RNA from FEMX-I cells and exosomes was prepared with Qiazol extraction followed by poly-A tailing reactions and miRNA cDNA synthesis. 250 ng of cell’s total RNA and of exosomes’ RNA were used in cDNA synthesis. cDNA synthesis were carried out simultaneously and equal volume of cDNA synthesis reaction product was used in the subsequent real-time qPCR reactions. 1058 miRNAs were investigated. The DeltaCt (DCt) values for each miRNA-specific prom1-exo cDNA greater than 0.01 were listed.

### Specific “loading” of miRNAs in prom1-exo

The miRNA “cargo” of prom1-exo was significantly different from the parental cell content. Of the 1,058 miRNA species investigated, only 49 were over-expressed in prom1-exo (Table 3), including miRNAs known to mediate immune tolerance, and 13 cancer/metastasis-associated miRNAs. In particular, miR-216b, a well-known tumor and metastasis suppressor mi-RNA, that targets Ras [43,44], was highly expressed in prom1-exo and undetectable in FEMX-I cells, indicating a detoxification role for prom1-exo; let-7i, associated with metastatic progression [45-47], was found to be expressed at levels 53-fold higher in prom1-exo than in FEMX-I cells. Also, miR-10a, reportedly involved in the metastatic process and immune-escaping [48,49] was 3.2-fold higher in prom1-exo than in parental cells.

### Transfer of prom1-exo to adjacent FEMX-I and MSC

**Figure 6:**
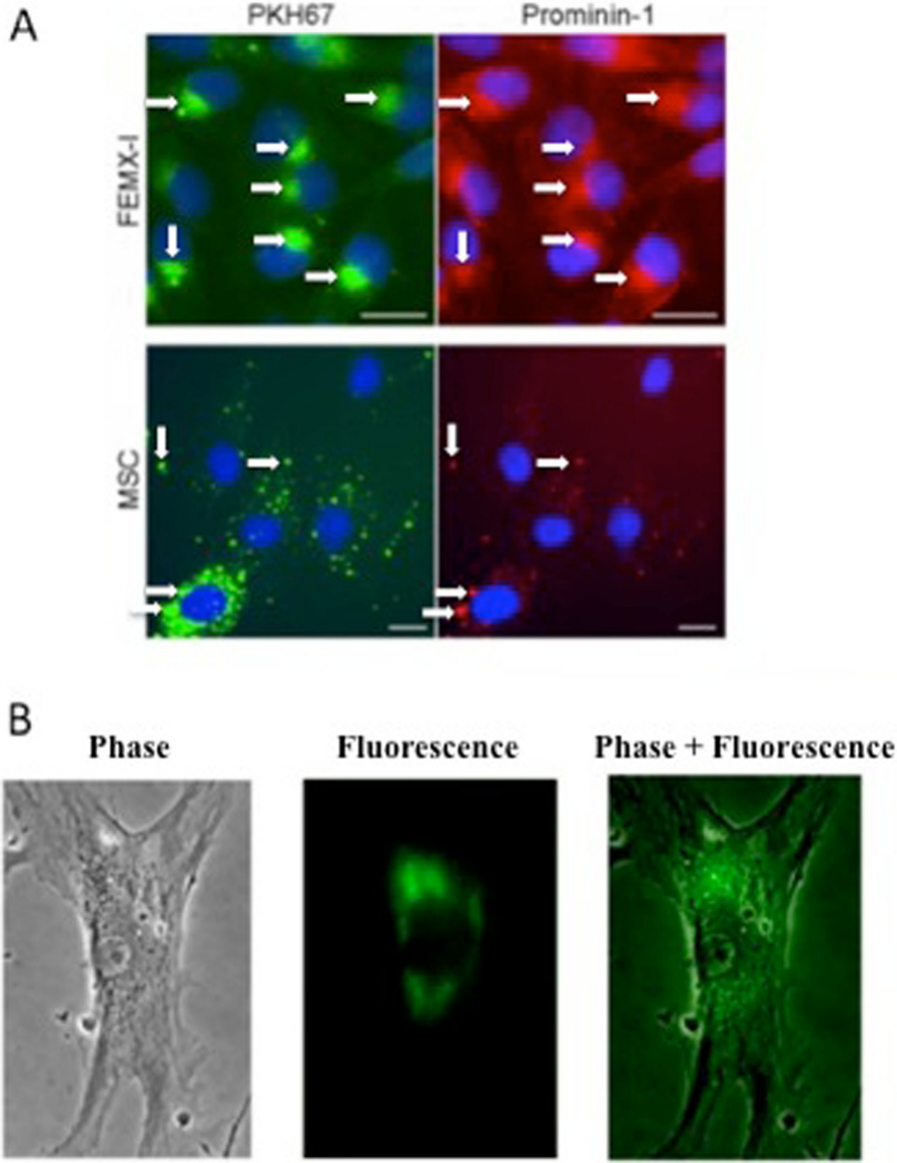
**Uptake of prom1-exo by FEMX-I and MSC. A**. Cells were incubated for 3 h with PKH67-labeled green fluorescent prom1-exo, fixed and stained with phycoerythrin-conjugated AC133 anti-prominin-1 antibody. Arrows represent areas of co-localization of green PKH67 fluorescence and red fluorescent anti-prominin-1 antibodies. Since MSC do not express prominin-1, there could be no interference from an endogenous MSC prominin-1 pool. Bars, 25 μm. **B**. MSC were incubated for 3 h with prom1-exo prepared from FEMX-I cells transiently transfected with a prominin-1-GFP fusion plasmid.

**Figure 7:**
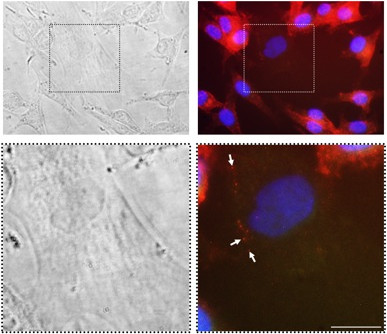
**Co**-**culture of MSC and FEMX-I cells shows uptake of prominin-1 by MSC.** MSC and FEMX-I cells were cultured for 24 h at 1:5 ratio. After fixation and permeabilization, expression of prominin-1 was analyzed by immunofluorescence employing phycoerythrin-conjugated AC133 anti-prominin-1 antibody. Since MSC do not express prominin-1, there could be no interference from an endogenous MSC prominin-1 pool. Insets in the upper panels were enlarged in the lower panels. Arrows indicate some areas of prominin-1 positivity inside a MSC. Red, prominin-1; blue, DAPI. Bars, 25 μm.

Exposure of FEMX-I cells to PKH-67-labeled prom1-exo for 3 h resulted in massive green perinuclear fluorescence (Figure 6A), co-localized with the red fluorescence of the intracellular pool of prominin-1 upon incubation of the cells with phycoerythrin-conjugated monoclonals (Figures 6A and 3A-B). Interestingly, also exposure of human MSC to PKH-67-labeled prom1-exo for 3 h resulted in intra-cellular localization of fluorescent prom1-exo and of prom1-exo-associated prominin-1 (Figure 6A); for MSC, the punctate pattern differed from the perinuclear accumulation for FEMX-I cells, presumably for the lack of an endogenous pool of prominin-1 in MSC. Since a shorter (1 h)-exposure of FEMX-I to PKH-67-labeled prom1-exo resulted in exclusive, although minor, perinuclear accumulation of green fluorescence (data not shown), the complete absence of puncta in FEMX-I cells may be due to the rapid kinetics of intracellular exosome trafficking or turnover. To confirm the intracellular delivery of prominin-1 by prom1-exo, MSC were incubated with prom1-exo prepared from FEMX-I cells transiently transfected with a prominin-1-GFP fusion plasmid. After 3 h, extensive fluorescence from prominin-1-GFP was detected in the intracellular compartment of MSC (Figure 6B), confirming that prominin-1 was effectively delivered to MSC. To investigate whether direct transfer of prom1-exo occurred from FEMX-I to MSC in mixed cultures, we co-cultured the cells at 5:1 ratio (FEMX-I:MSC) for 24 h, and analyzed the expression of prominin-1 by immunofluorescence. Figure 6 clearly shows transfer of prominin-1 from FEMX-I to the intracellular compartment of MSC. The apparent contrast between the massive uptake of exosomes in Figure 6 and the relatively low transfer of exosomes from FEMX-I to MSC in Figure 7 may be explained by the technical differences of the two experiments (sudden addition of exosomes in Figure 6 and gradual release of exosomes in Figure 7).

### Effects of exosomes on MSC invasiveness

**Figure 8:**
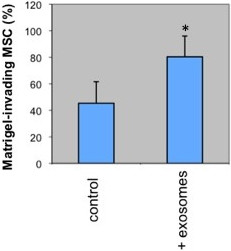
**Enhanced invasiveness of MSC through matrigel induced by prominin-1-purified exosomes.***In vitro* invasion assays were performed in BioCoat invasion chambers holding matrigel-coated-8 μm-pore PET membrane cell culture inserts, using non-coated inserts as control. The lower chambers were filled with medium containing 2% FBS, and equal aliquots of MSC, pre-incubated with or without prominin-1-purified exosomes, were added in serum-free medium to the inserts. Following 24 h incubation at 37°C, as recommended by the manufacturer, the cells on the upper side of the membrane were gently removed with wet cotton swabs. The cells on the lower surface of the membranes were fixed with 4% para-formaldehyde for 10 min, and then stained with DAPI. Matrigel invasiveness is expressed as the percentage of the number of matrigel-invading cells respect to the control of chemotactic migration. Columns, mean values of three separate experiments; bars, SD; *p < 0.05, unpaired Student’s t test.

We then investigated whether exposure of MSC to prom1-exo resulted in biological effects, such as changes in their invasiveness, measured by the capacity of MSC to pass through a Matrigel layer. Interestingly, a 90% increase in Matrigel invasion was observed after a standard 24 h-assay, compared with mock-treated MSC (Figure 8).

## Discussion

Building on our previous finding of a pro-metastatic role of prominin-1 in melanoma [9], we have here isolated from *in vitro* serum-free cultures of FEMX-I melanoma prominin-1-expressing exosomes. This is the first report of prominin-1-based purification of cancer exosomes. The prominin-1-based exosomal isolation protocol was then successfully employed to isolate exosomes from the serum-free culture medium of another prominin-1-expressing cancer cell line, Caco-2 colon carcinoma. Interestingly, Tauro *et al*. [50] recently reported the isolation, via sequential immunocapture using anti-A33- and anti-EpCAM-coupled magnetic beads, of prominin-1-expressing exosomes from the human colon carcinoma cell line LIM1863.

Consistent with findings of other groups in different experimental models [51,52], a specific sorting of proteins, lipids and microRNA was observed in in prom1-exo. Preparations of exosomes from biological fluids and *in vitro* cell cultures using a variety of strategies and techniques have been extensively reported by many groups; however, the great majority of preparations contain varying proportions of other membranous vesicles that co-purify with exosomes, such as shed microvesicles and apoptotic blebs [8,33]. The importance of analyzing purified exosomal preparations is evident if we consider that although exosomes, due to their small size (40–100 nm), are expected to contain less than 150 proteins, to date over 4,500 proteins have been identified in exosomes from multiple organisms (http://exocarta.org). While some of these proteins are considered tissue-specific, many can be considered preparation contaminants. The fact that prom1-exo present respectively a 78- and 168-fold higher concentration of prominin-1 and alix compared with microvesicles derived from the same cell line, as well a striking concentration of certain classes of microRNA, indicates that prom1-exo constitute an homogeneous species of exosomes, loaded with a pro-metastatic cargo.

It is now becoming clear that, when a complex message needs to be sent to surrounding cells in the microenvironment, cells use exosomes, which have the advantage, compared to other means of intercellular communication, to target multiple specific locations inside the target cell(s), based, at least in part, on the specific Rab proteins expressed, which act as mailing tags to distribute exosomes to the correct intracellular compartment. The presence of 5 distinct Rabs (Rab 5B, Rab 5C, Rab 7A, Rab 8A and Rab 10) suggests that prom1-exo are destined to different endosomal compartments in the host/target cell. For example, Rab 5C, 7A and 8A are indispensable effectors/constituents of early endosomes, late endosomes, and secretory endosomes, respectively [53,54]. Herein, we have clearly shown rapid uptake of prom1-exo into neighboring FEMX-I cells and MSC, associated with intracellular delivery of prominin-1. Our finding that prom1-exo contain proteins involved in the ESCRT complex suggests that, once they reach their target cell(s), prom1-exo are able to be endocytosed into the endosomal system of recipient cells and deliver their “cargo” into the cytoplasm as a reversal mechanism of their formation process. Interestingly, the presence in prom1-exo of the “fusogenic” proteins CD9, CD63, CD81, ADAM 10, GTP-binding protein α13 and RhoA [55-59] suggests also an alternative mechanism of “cargo” delivery, i.e. receptor-mediated fusion with the plasma membrane of the host cell(s).

The presence of both syntenin-1 and alix in prom1-exo supports a recent theory of the biogenesis of intraluminal vesicles (ILVs) and exosomes [60], while the presence of two heat shock proteins, hsp70 and hsc70 and of the hsc-70 co-chaperone dj2 [61] is in agreement with their function in endosomal cargo selection and in general as molecular chaperones, limiting protein aggregation, and facilitating protein refolding. Our data are consistent with the previous finding of secretion of heat shock proteins into the circulation via lipid raft-mediated, or exosome-mediated exocytosis in tumor cells [62]. Interestingly, the high level of expression of IgSF8, known to have an immunosuppressor role, by inhibiting T-cell mobility coordinately with CD81 [63], and the finding by other groups that extracellular HSPs exert immunomodulatory activities [62] suggest an important role of prom1-exo in the immune escape of FEMX-I melanoma.

Several tetraspanins were identified in prom1-exo. Many members of the tetraspanin family, the most abundant protein family found in exosomes [33], including most of the tetraspanins present in prom1-exo, regulate cell migration, fusion, and signaling events by their recruitment into special membrane microdomains and their abundant presence in microvesicles that mediate intercellular communication [64]. Interestingly, tetraspanins organize other proteins through intra- and inter-molecular interactions into a multimolecular tetraspanin enriched microdomains (TEMs), which, in association with other proteins and lipids, such as cholesterol and sphingomyelin [65], forms the extended network of tetraspanin interactions in the membrane, commonly described as tetraspanin web [66]. Our findings of co-expression of many tetraspanins and of a 4-fold increase in sphingomyelin supports the concept that tetraspanin webs are building blocks of prom1-exo.

By ESI/MS-MS lipid profiling, we found that typical raft components were associated with prom1-exo. The fact that other cancer cell types secrete exosomes containing similarly organized lipid subdomains [51,67], suggests that lipid rafts may play a general role in exosome biogenesis and structure, especially sphingolipids, known to play a key role in the genesis of exosomal MVBs [68], and phosphoglycerides with long and saturated fatty-acyl chains [67-69]. Our data also support the hypothesis that the lipid raft composition of endosomes, of which exosomes represent an extracellular mirror, allows them to be multi-purpose platforms [70].

As proposed for hematopoietic and neural stem cells [71,72], prominin-1 may have a specific role in intercellular communication via exosomes, and protein–lipid assemblies might be the essential structural determinant in the release process of prominin-1 by stem and cancer stem cells. In addition, the full molecular characterization of prom1-exo described here supports the concept of ‘cancer stem cell-specific lipid rafts’ holding molecular determinants necessary to maintain cancer stem cell/pro-metastatic properties [72]. Interestingly, the high sphingomyelin and phosphatidylserine content of prom1-exo may lead to their capacity to fuse with the plasma membrane of host cells and enter the intracellular compartment [32]. In fact, lipid rafts reportedly [73] affect protein binding and modulate membrane physicochemical and mechanical properties: thus, sphingomyelin-enriched microdomains modulated the efficiency of membrane fusion [74], and annexin V blockade of phosphatidylserine on the surface of exosomes prevented exosome uptake into microglia [75].

The 21.5-fold increase in lyso-phosphatidylethanolamine observed in prom1-exo may contribute to the pro-metastatic phenotype of FEMX-I cells, in light of the report by Park et al. [76] that lyso-phosphatidylethanolamine treatment of SK-OV3 ovarian cancer cells results in chemotactic migration and cellular invasion. However, whether this mechanism or the transfer of metalloproteinases, such as ADAM10, present in prom1-exo, is responsible for the increased invasiveness of MSC upon exposure to prom1-exo can not be concluded from the present study.

Consistent with other previous studies [77-79], a considerable difference in the miRNA profile of cancer exosomes and the originating cancer cells was observed in the present study. Specifically, 49 miRNA were found to be over-expressed in prom1-exo, including miRNAs known to mediate immune tolerance, and 13 cancer/metastasis-associated miRNAs. This is in apparent contrast with the claim from several authors [80,81] that the miRNA content of circulating exosomes is similar to that of the originating cancer cells. The cancer-associated loss of miRNA expression often leads to a proliferative advantage and aggressive behavior through largely unknown mechanisms. The finding of very high levels of miR-216b in prom1-exo, coupled with undetectable levels in parental FEMX-I cells, is intriguing in light of reports that miR-216b suppresses tumor growth and invasion by targeting KRAS in nasopharyngeal carcinoma [43] and inhibits cell proliferation and colony formation through Ras inhibition in a pancreatic cancer model [44]. Similarly, a 53-fold lower level of let-7i was observed in FEMX-I cells compared with prom1-exo. Since underexpression of let-7i was found to characterize metastatic progression of oral carcinoma [45] and to have a crucial role in colorectal cancer metastasis [46], it is conceivable that exosomal removal of both miR-216b and let-7i from the intracellular compartment plays a significant role in the malignant phenotype of FEMX-I melanoma. While removal of some species of microRNAs may have a detoxification role, exosomal delivery of other species of microRNA, such as miR-10a, to other cells in the microenvironment may play an important role in FEMX-I melanoma immuno-escape. In fact, miR-10a, present in prom1-exo at levels 3.2-fold higher than in parental cells, was recently shown to attenuate the phenotypic conversion of inducible T(reg) cells into follicular helper T cells and limit differentiation into the T(H)17 subset of helper T cells [48]. Also, miR-10a reportedly stimulates cell invasion, suggesting a potential mechanism for the pro-invasive effect of prom1-exo on MSC [49]. Therefore, prom1-exo may accomplish for FEMX-I melanoma cells a double role of cell detoxification via excretion and of modulation of the function of other cell types, in particular MSC, in the microenvironment. Our data, suggesting a pro-malignant role of prom1-exo, are consistent with a recent report by Peinado et al. [7] that exosomes from highly metastatic melanomas increased the metastatic behavior of primary tumors by permanently ‘educating’ bone marrow progenitors through the receptor tyrosine kinase MET. To metastasize, tumor cells need to send complex messages intended to subvert the normal function of their immediate neighbors, fertilize vasculogenesis and find or recruit a susceptible berth. However, messages of all sorts are being identified in many functional exosomal studies, and if we sample them stochastically it will be difficult to see the whole picture. Prom1-exo, homogenous cancer organelles expressing a cancer stem cell marker, are more likely to have a concordant message(s), and this makes them especially interesting to gain insight into the mechanisms by which exosomes contribute to the malignant phenotype. In addition, our characterization of prom1-exo from FEMX-I cells may be employed as a model for investigating the rules that govern the formation of membrane microdomains: in contrast to rafts, exosomes are remarkably stable structures that can be purified without the intervention of destructive techniques such as detergents or ultrasounds. Our model, therefore, in addition to allowing progress in the understanding of the role(s) of cancer-derived exosomes in the metastatic process, can also shed light on the natural process of selective proteolipidic sorting in biological membranes and trafficking in living cells. Further studies are warranted to determine what part of their cargo and which molecular mechanisms exosomes, and in particular prom1-exo, utilize to modify the phenotype of the different cells in the local tumor microenvironment and exert specific roles in the metastatic phenotype.

## Abbreviations

Prom1-exo: Prominin-1-expressing exosomes; MSC: Bone marrow-derived stromal cells; NTA: Nanoparticle tracking analysis; ESI: Electrospray ionization; TEM: Tetraspanin enriched microdomain; MVB: Multivesicular body; VPS: Vacuolar protein sorting; ESCRT: Endosomal sorting complex required for transport.

## Competing interests

The authors declare that they have no competing interests.

## Authors’ contributions

GR and AL designed and executed most of the experiments and wrote the manuscript. JM analyzed proteins by Western blotting and performed the invasion assays. FA prepared microvesicles and exosomes. RMP analyzed proteins by mass spectrometry, wrote the proteomics part of the manuscript and helped writing the manuscript. All authors read and approved the final manuscript.

## Supplementary Material

Additional file 1: Table S1 Search conditions for proteomic LC-MS/MS data sets. MS/S data was acquired during 70 min gradients run on hand-packed capillary columns as described in Materials and Methods. The effluent was interfaced to an ESI source and peptides were recorded with data-dependent scanning using a top 5 method on an LTQ/XL ion trap (Thermo). Table 1 describes data processing, search conditions common to both the MASCOT and Spectrum Mill Proteomics Workbench applied in this work as well as particular features of the SwissProt database used. Click here for file

Additional file 2: Table S2 Protein assignments. Use of the Peptide and Protein Prophet algorithms to condense independent searches of the same data sets provided 282 proteins (including keratins) across a total of three biological isolations of prom1-exo with an FDR of 0.1%, a minimum of three peptides and a protein sensitivity of 99%. Additional file 2 lists proteins matching these criteria. Click here for file

Additional file 3: Table S3 All Peptides attributed to proteins from Additional file 2. All peptides identified in three replicate isolations of prom1-exo. Scaffold 4.0.0 was used to rescore the results of MASCOT and SpectrumMill searches. Scaffold generates an adaptive discriminate scoring using Peptide and Protein Prophet algorithms. Complete results are listed in Additional file 3: Table S3. Click here for file

Additional file 4: Table S4 Selection of the most observable proteins associated with prom1-exo. Tables 1 and 2 of the manuscript illustrate protein enrichment for physiological processes involving endosome and ESCRT complexes. These proteins are highlighted among an ensemble verified with two or more stringent peptide spectral matches (PSM) in all three replicates or those observed with three or more stringent PSM in any two replicates. Additional file 4: Table S4 is a complete list of these 154 proteins. Click here for file

Additional file 5: Figure S1 Enrichment for physiological processes of the most observable proteins associated with prom1-exo. Click here for file
