# Supplementary figures and images for: Biochemical and biological characterization of exosomes containing prominin-1/CD133
Source: Mol Cancer. 2013 Jun 14;12:62. doi: 10.1186/1476-4598-12-62 (PMC3698112; doi:10.1186/1476-4598-12-62)

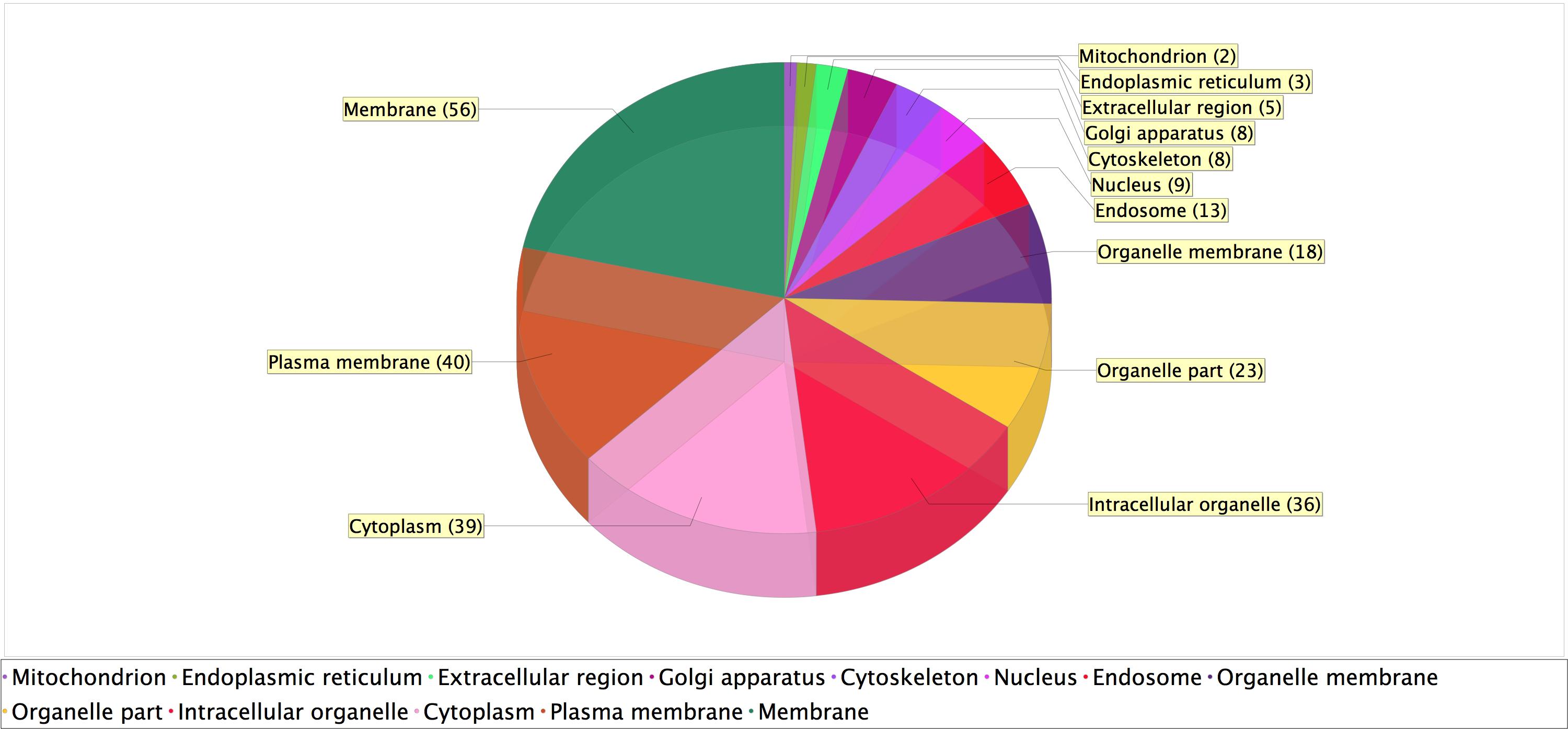

Supplement: Additional file 5: Figure S1 — Enrichment for physiological processes of the most observable proteins associated with prom1-exo. [file 1476-4598-12-62-S5.jpeg]
